# Population pharmacokinetic analysis of remimazolam after continuous infusion for sedation in critically ill patients

**DOI:** 10.3389/fphar.2025.1526266

**Published:** 2025-07-09

**Authors:** Jingchun Chen, Xipei Wang, Dong Chen, Xiaolong Liu, Kaiyi Peng, Ruizheng Tang, Linhui Hu, Yirong Wang, Yunpeng Bai, Lin Chang, Chunbo Chen

**Affiliations:** ^1^ Department of Critical Care Medicine, Shenzhen People’s Hospital (The First Affliated Hospital, Southern University of Science and Technology; The Second Clinical Medical College, Jinan University), Shenzhen, China; ^2^ School of Medicine, South China University of Technology, Guangzhou, China; ^3^ Department of Critical Care Medicine, Guangdong Provincial People’s Hospital (Guangdong Academy of Medical Sciences), Southern Medical University, Guangzhou, China; ^4^ Research Center of Medical Sciences, Guangdong Provincial People’s Hospital (Guangdong Academy of Medical Sciences), Southern Medical University, Guangzhou, China; ^5^ Guangdong Provincial Key Laboratory of Clinical Pharmacology, Guangdong Provincial People’s Hospital (Guangdong Academy of Medical Sciences), Southern Medical University, Guangzhou, China; ^6^ Department of Critical Care Medicine, Maoming People’s Hospital, Maoming, China; ^7^ Department of Pharmacy, Shenzhen People’s Hospital, The Second Clinical Medical College of Jinan University, The First Affiliated Hospital of Southern University of Science and Technology, Shenzhen, China; ^8^ Yichang Humanwell Pharmaceutical Co. Ltd., Yichang, China

**Keywords:** critically ill patients, population pharmacokinetics, remimazolam, sedation, context-sensitive decrement times

## Abstract

**Introduction:**

The aim of the present prospective study was to model the population pharmacokinetics of remimazolam after continuous infusion in critically ill patients, and to provide a guide for remimazolam administration based on simulations that were conducted.

**Patients and methods:**

A total of 32 critically ill patients were enrolled in this study, with 236 plasma concentration data ultimately included for modeling. Plasma concentrations of remimazolam were quantified by a validated high-performance liquid chromatography-tandem mass spectrometry method, and the data were analyzed using non-linear mixed effect modeling. Concentration-time curves of remimazolam at different induction and maintenance doses were simulated and context-sensitive decrement times (CSDTs) were calculated using Monte Carlo simulations.

**Results:**

A two-compartment model appropriately described the concentration-time profile of remimazolam in critically ill patients. The elimination clearance, volume of the central compartment, volume of the peripheral compartment, and peripheral compartmental clearance were estimated to be 58.2 L/h (95% CI, 47.8–72.3 L/h), 25.5 L (95% CI, 16.8–33.3 L), 34.5 L (95% CI, 26.0–58.8 L) and 21.9 L/h (95% CI, 12.2–34.6 L/h), respectively. No covariates significantly influenced the pharmacokinetic parameters of remimazolam. Internal validation proved the reliable predictive performance of the model. The CSDTs of remimazolam (10%–90%) was independent of the infusion time.

**Conclusion:**

Remimazolam showed a predictable pharmacokinetic profile and was demonstrated to be suitable for long-term sedation in the intensive care unit, with dose adjustments only required dependent on the degree of the sedative effect.

## 1 Introduction

Sedation is an indispensable treatment strategy in the ICU, but nevertheless excessive or inadequate sedation increases morbidity, mortality and resource consumption (Kumpf et al., 2023). Currently, ICU sedation complies with the eCASH concept which aims to establish optimal patient comfort with minimal sedation (Vincent et al., 2016). The ideal sedative agent is required to be characterized by producing a low incidence of adverse effects, predictable recovery, rapid conversion to inactive metabolites, non-dependence on organ function, and non-accumulation in tissues during the maintenance of general anesthesia (Kim and Fechner, 2022). Remimazolam is a novel sedative drug with pharmacokinetic properties suggested that it might be of great potential to utilize for the sedation of critically ill patients.

Remimazolam is a short-acting benzodiazepine derivative developed from a combination of remifentanil and midazolam properties, having a metabolizable methyl propionate side-chain structure similar to remifentanil, and a mechanism of action similar to midazolam (Kilpatrick et al., 2007; Hu et al., 2022). It exerts it actions by interacting with the gamma-aminobutyric acid type A (GABAA) receptor, increasing the flux of chloride ions, causing membrane hyperpolarization and inhibition of neuronal activity, resulting in reduced organismal activity, sedation, amnesia, *etc.* Of note is that these effects can be easily antagonized by administering flumazenil (Stafford, et al., 2002; Jacob et al., 2008). It was demonstrated that remimazolam is metabolized by carboxylesterase-1 (CES-1) to produce zolpidem propionic acid, which has <1/300 times the affinity of remimazolam for the GABAA receptor and has minimal pharmacological activity (Freyer et al., 2019; Sneyd and Rigby-Jones, 2020; Stöhr et al., 2021). In healthy volunteers (Antonik et al., 2012), remimazolam exhibited linear pharmacokinetic characteristics in the dose range of 0.01–0.30 mg/kg, with a steady-state volume of distribution (V_d_) of 34.8 ± 9.4 L, a clearance (CL) of 70.3 ± 13.9 L/h, and a time of terminal half-life of 45 ± 9 min. A model (Masui et al., 2022) that included data from patients anesthetized for more than 7 h demonstrated that the context-sensitive half-time (CSHT) of remimazolam was independent of the infusion time of 360 min. Therefore, remimazolam is characterized by a rapid onset of action, rapid recovery and no drug accumulation.

Currently, remimazolam has been mainly administered for procedural sedation and general anesthesia, while its application in the ICU are still at the stage of clinical trials. Zhou et al. (2020) investigated factors influencing the variability in the remimazolam response in general anesthesia and found that lower doses could be considered in elderly patients with a serious disease burden or ASA class 3 patients, whereas Stöhr (Stöhr et al., 2021) found that CL was 38.1% lower in subjects with severe hepatic impairment compared to healthy volunteers. However, for critically ill patients with complex and severe pathophysiologic conditions, the pharmacokinetic parameters of the drug may be altered, and consequently affect its pharmacodynamics (Wang et al., 2021; 2023; Herten et al., 2022). In addition, many critically ill patients suffer from cardiac, pulmonary or renal dysfunction, requiring the use of extracorporeal membrane oxygenation (ECMO) or continuous renal replacement therapy (CRRT), which have the potential to impair drug exposure (Lan et al., 2022; Wu et al., 2022). A clinical trial of mechanical ventilation in an ICU in Japan reported (K.U., 2017) that seven patients who were administered the drug for more than 24 h had higher than expected plasma concentrations of remimazolam. The results of the latter study suggested that prolonged drug administration could be a challenge for ICU medication usage (Wiatrowski et al., 2016). To date, as far as we are aware, there are no published reports on the pharmacokinetic characteristics of remimazolam administered to ICU patients. In order to evaluate further its potential effects and adverse events during ICU sedation, it is crucial to investigate the pharmacokinetic properties of remimazolam in this population of patients.

The aim of this prospective and observational study was to model the population pharmacokinetics (PopPK) of remimazolam after continuous infusion in critically ill patients, to investigate covariates that may affect its pharmacokinetic properties, and to provide a guide for remimazolam administration based on a dosing simulation.

## 2 Patients and methods

### 2.1 Patients and setting

This study was conducted from April 2022 to December 2022 in the Department of Critical Care Medicine, Maoming People’s Hospital. The research was performed according to the World Medical Association Declaration of Helsinki and approved by the local ethics committee of Maoming People’s Hospital (No. PJ2021MI-K009-01). Patients who received remimazolam for sedation in the ICU were prospectively recruited to the study. The inclusion criteria were: (i) patients aged from 18 to 85 years who were administered remimazolam through an intravenous micropump; and (ii) patients or guardians gave acceptance for multiple blood collections during the study. Patients who had allergic reactions to remimazolam or incomplete clinical information were excluded. All patients or their legally authorized representatives provided written informed consent for the procedures.

### 2.2 Dosing and data collection

A final concentration of one or 2 mg/mL remimazolam besylate (Yichang Humanwell Pharmaceutical Co., Ltd., Hubei, China) was prepared by diluting 50 mg or 100 mg of the drug in 0.9% saline (50 mL). The schematic chemical structure and major physicochemical properties of remimazolam have been listed in Supplementary Figure S1; Supplementary Table S1. Remimazolam was administered through an infusion micropump, with the pump speed modified to achieve an acceptable degree of sedation. The degree of sedation was scored by the clinical nurse *via* the Richmond Agitation-Sedation Scale (RASS), and the clinical decisions on the use and dosing regimens of remimazolam were undertaken by the clinician responsible for each patient.

Demographic data, including sex, age, height, weight, reason for ICU admission, length of hospitalization and ICU stay, clinical outcomes, and causes of death, were carefully recorded for all patients. Laboratory test data included: (1) hepatic and renal function indicators—alanine aminotransferase (ALT), total protein (TP), albumin (ALB), and serum creatinine (SCR); (2) inflammatory and hematologic markers—C-reactive protein (CRP), leukocyte count (WBC), platelet count (PLT), and hemoglobin (Hb), all measured daily following medication administration. Creatinine clearance (CrCL) was calculated using the Cockcroft-Gault formula, and the glomerular filtration rate (GFR) was estimated using the Chronic Kidney Disease Epidemiology Collaboration (CKD-EPI) formula. Acute Physiology and Chronic Health Evaluation (APACHE) II scores were documented on the day of ICU admission or drug administration. For patients undergoing procedures with ECMO or CRRT, various parameter values and the length of treatment were recorded.

### 2.3 Sample collection and measurements

Blood samples from critically ill patients were collected in K2-EDTA tubes at the time of dose cessation and 10, 20, 30, 60, 90, 120 and 240 min after discontinuation of dosing (Sheng et al., 2020; Brum, 2022). Plasma samples were obtained by centrifuging the blood samples (996 g, 10 min, 4°C) and then storing them at −80°C at the Biological Resource Center before subsequent analysis.

All concentrations of remimazolam in plasma were quantified using a validated high-performance liquid chromatography-tandem mass spectrometry (HPLC-MS/MS) analytical method (Chen et al., 2023) at the central laboratory of Maoming People’s Hospital. The internal standard (IS) was carbamazepin-d8 and the plasma sample extraction method was protein precipitation. The HPLC system (Agilent 1260) used a ACQUITY UPLC^®^ BEH C18 column (Waters, 2.1 × 50 mm, 1.7 μm) coupled to a 6460 electrospray ionization-triple quadrupole mass spectrometer. Elution was performed using mobile phase A (10 mM ammonium acetate in water containing 0.2% formic acid) and mobile phase B (acetonitrile) under a gradient program, with retention times of 2.00 and 1.43 min for the analyte and IS, respectively. The ion pairs of remimazolam used for quantification were m/z 439.2 → 407.1 and m/z 245.2 → 202.2, with a linear range was 1.0–1000 ng/mL. The intra- and inter-batch precision were both ≤2.17%, with an accuracy between 95.5% and 102.2%.

### 2.4 Population pharmacokinetic model development

We analyzed the remimazolam concentration data using non-linear mixed effects modeling software NONMEM 7.3 (version 7.3, ICON plc, NY, USA). The first-order conditional estimation with inter- and intra-subject variability interaction was applied for model parameter estimation. One-, two- and three-compartments were tested to fit the plasma concentration-time data of remimazolam and to screen the appropriate base model. All covariates that could potentially influence the pharmacokinetic parameters of remimazolam were evaluated, including demographics such as age, sex, and weight, hepatic and renal function indicators such as SCR, ALT, and TP, as well as the use of CRRT or ECMO. Categorical variables (such as sex) were modeled as follows:

$$\theta_{ij}=\theta_{tv,j}\cdot \theta_{j}^{COV}\cdot e^{\eta_{i}}$$

In the case of continuous covariates (such as age), an exponential model with median covariate values and adjustment factors was employed thus:

$$\theta_{ij}=\theta_{tv,j}\cdot {\left(\frac{COV}{{COV}_{Median}}\right)}^{\theta_{j}}\cdot e^{\eta_{i}}$$

In the above formula, *θ*_*ij*_ is the individual parameter for each patient, *θ*_*tv,j*_ is the typical value of the parameter, *θ*_*j*_ is an impact factor and COV is the value of the covariate (when COV was a categorical variable, taken to be 0 or 1). *η*_*i*_ represented inter-individual variability (IIV), which was normally distributed with a mean of 0 and variance *ω* ^*2*^. Residual variability (RV) was modeled by a mixed model of additive and proportional errors, which reflected the level of random variation in the predicted values with respect to the observed values.

The objective function values (OFV) of the model were an indicator of the goodness of fit, where the variance met the distribution of $\chi^{2}$ with the approximate degree of freedom (*df*). For covariate screening, forward stepwise univariate analysis was used with a decrease of OFV of at least 3.84 (α = 0.05, *df* = 1). A backward elimination analysis of the covariates increased of OFV by at least 10.82 (α = 0.001, *df* = 1). The decrease in IIV and RV values were also investigated.

### 2.5 Population pharmacokinetic model diagnostics

The diagnostic plots, bootstraps, prediction-corrected visual predictive check (pcVPC) and normalized prediction distribution error (NPDE) were used to comprehensively evaluate the models in this study.

Prediction-based model diagnostic plots illustrated the goodness of fit in a model by presenting the conformance between the observation and the population prediction (PRED) or the individual prediction (IPRED). Diagnostic plots of conditional weighted residuals (CWRES) vs time or PRED were used to estimate the prediction error. The non-parameter bootstraps were resampled 1,000 times to generate the datasets which were used to assess the accuracy and the stability of the model estimates. The distributional characteristics of the observed and the simulated data were graphically displayed in pcVPC. NPDE was based on 1,000 simulations and the model evaluated using the Wilcoxon signed rank test, Fisher’s variance test or Shapiro-Wilks test when appropriate.

### 2.6 Simulations

Monte Carlo simulations were conducted using the final model to generate data from 1,000 subjects to investigate the relationship between the remimazolam dose and the plasma concentration. In combination with covariates, concentration-time curves were calculated under different loading doses (3–8 mg/min) of 1-min duration and maintenance doses (6–18 mg/h) of up to 16 h. Context-sensitive decrement times (CSDTs) for remimazolam were estimated from population predicted values. The time to 10%–90% decrement in the concentrations of remimazolam between 0.5 h and 72 h by continuous infusion at a maintenance dose of 12 mg/h were simulated.

### 2.7 Statistical analysis

Baseline data were expressed as frequencies (percentage) for discrete variables. Continuous variables were presented as median (minimum-maximum) and interquartile range. R software (version 4.2.2) was used for statistical analysis and graphical plotting. The analysis procedure was run through Pirana (version 2.9.7). Bootstrap and pcVPC were run on Perl-speaks-NONMEM version 4.8.0 (Uppsala University, Sweden).

## 3 Results

### 3.1 Patient characteristics

**FIGURE 1:**
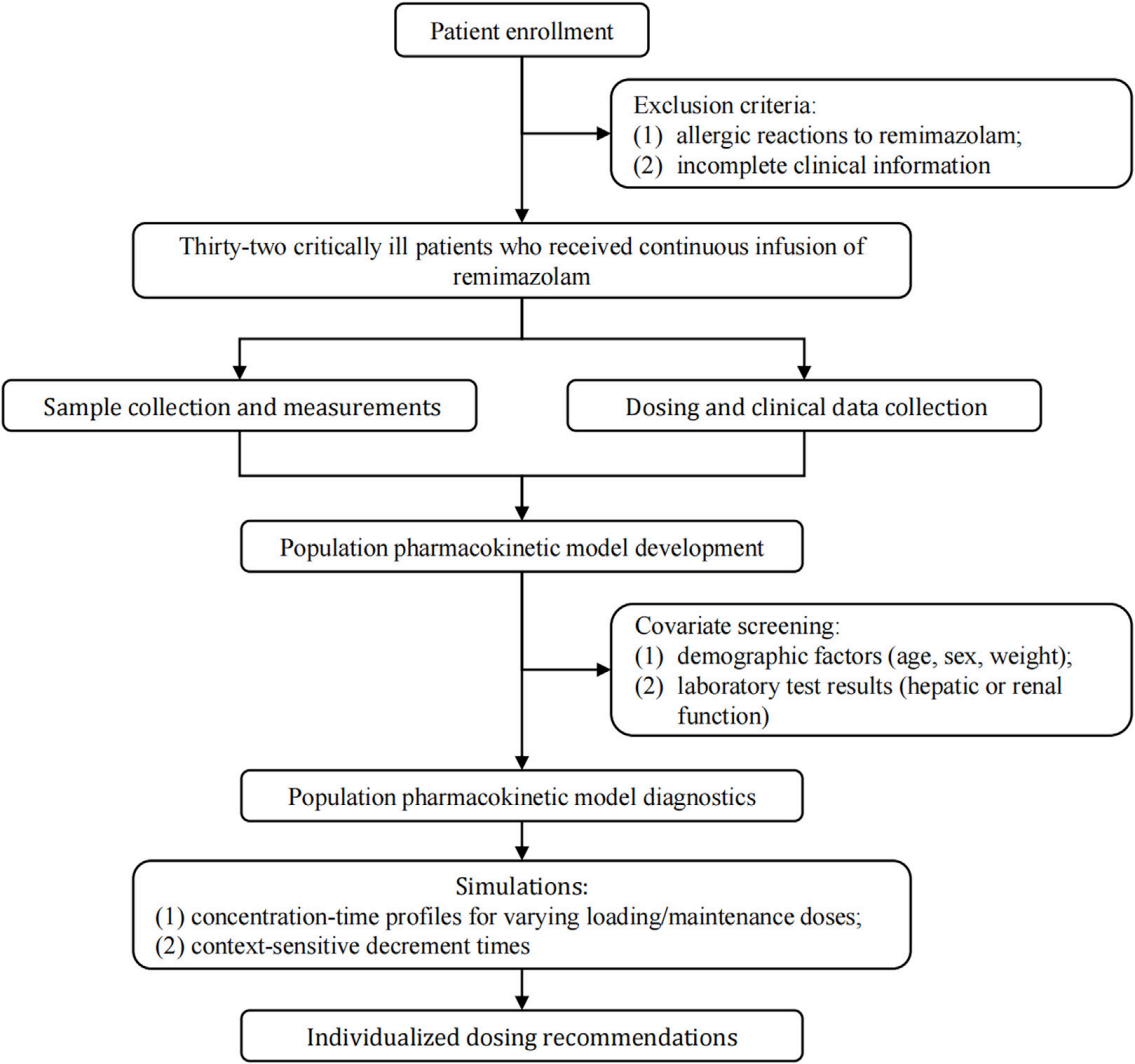
Study framework.

**FIGURE 2:**
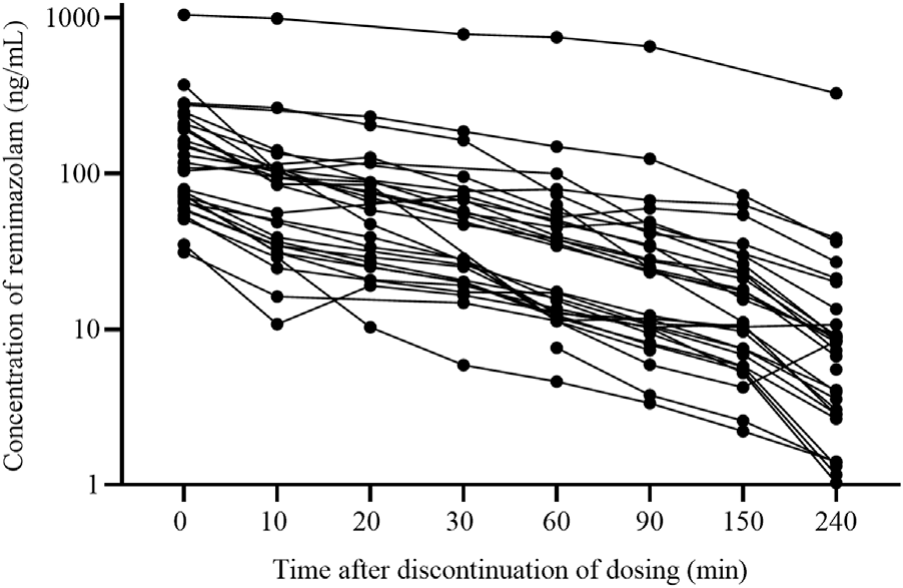
The plasma concentration-time curves of remimazolam after discontinuing infusion (n = 32).

Figure 1 showed the protocol and flow of the PopPK study of remimazolam. A total of 32 patients (8 females, 24 males) were enrolled in the study. A total of 243 plasma samples were collected, seven of which were discarded due to unreasonably higher or lower concentrations. The final dataset consisted of 236 plasma samples. The plasma concentration-time curves of remimazolam in each patient after discontinuation of the infusion were shown in Figure 2. The median age and BMI of the groups were 62 years and 22.67 kg/m^2^. All patients were administered remimazolam during the ICU stay and 13 of them (40.6%) received remimazolam immediately after admission to the ICU. The dosing regimen for remimazolam ranged from 2 to 17.28 mg/h, with the most commonly administered dosage being 6 mg/h. The median infusion duration of remimazolam was 8.33 h and the longest duration of administration 294.9 h. Most patients (96.8%) were co-administered of remifentanil for analgesia, and 15 patients were combined with sedative medications, including midazolam, dexmedetomidine, and propofol. Patients had normal to severely impaired renal and hepatic functions (CrCL 7.5–222.4 mL/min, ALT 7.8–549.2 U/L). Eighteen patients received ECMO and 14 underwent CRRT. The demographic characteristics of the patients are listed in Table 1.

**TABLE 1:** Demographic characteristics of the population (n = 32).

Characteristics	Value^a^	SD/IQR
Male/female, n (%)	24/8 (75/25)	NA
Age (year)	62 (26–79)	13.22
Body Weight (kg)	63 (47–98)	19.00
BMI (kg/m^2^)	22.67 (18.73–36.00)	3.45
APACHE II (on admission or sampling day)	26 (15–41)	7.89
Remimazolam dose (mg/h)	6 (2–17.28)	3.50
Remimazolam infusion duration (hours)	8.33 (6.15–294.9)	34.09
Biological results		
Alanine aminotransferase (U/L)	34.85 (7.8–549.2)	45.65
Total protein (g/L)	55.4 (26.82–85.29)	8.57
Albumin (g/L)	30.4 (15.97–51.7)	4.87
Total bilirubin (μmol/L)	20.49 (6.73–227.8)	18.55
Direct bilirubin (μmol/L)	8.2 (0.7–155.1)	12.27
Serum Cystatin C (mg/L)	1.28 (0.73–75)	1.45
Blood urea nitrogen (μmol/L)	6.65 (1.55–30.99)	8.52
Uric acid (μmol/L)	149 (22–1,118.6)	222.1
Serum creatinine (μmol/L)	75.75 (42.1–911.77)	101.75
C-reactive protein (mg/L)	95.68 (7.55–158.72)	60.37
Leukocyte (10^9^/L)	11.88 (2.03–39.04)	6.51
Platelet count (10^9^/L)	166 (15–611)	143.2
Hemoglobin	88 (47–141)	26.75
Hematocrit	27.25 (14.6–44.3)	2.82
Procalcitonin (ng/mL)	1.53 (0.1–66.3)	8.00
Arterial lactate (mmol/L)	1.6 (0.3–15)	1.25
Arterial Na (mmol/L)	137 (112–163)	45.65
Arterial K (mmol/L)	3.6 (2.7–5.6)	8.57
Arterial pH value	7.45 (7.04–7.73)	0.12
Kidney function		
Creatinine clearance (mL/min)	75.77 (8.69–193.83)	82.11
Estimated glomerular filtration rate (CKD-EPI) (eGFR, mL/min/1.73 m^2^)	86.90 (5.65–129.54)	73.56
Diagnosis of admission to ICU		
Respiratory failure	21 (28.7)	NA
Heart failure	19 (26.0)	NA
Severe pneumonia	11 (15.1)	NA
Septic shock	9 (12.3)	NA
Acute myocardial infarction	8 (11.0)	NA
COPD	5 (6.8)	NA
Combinational therapy		
Remifentanil, n (%)	31 (96.8)	NA
Dexmedetomidine, n (%)	4 (12.5)	NA
Butorphanol, n (%)	4 (12.5)	NA
Propofol, n (%)	2 (6.25)	NA
ECMO type and parameters		
ECMO (yes/no), n (%)	18/14 (56.3/43.7)	NA
Veno-arterial, n (%)	10 (55.5)	NA
Veno-venous, n (%)	7 (38.8)	NA
Veno-arterial-veno, n (%)	1 (5.55)	NA
Length of ECMO therapy (hours)	209 (52–568)	86
Blood flow (L/min)	3.3 (2–4.57)	0.648
Gas flow (L/min)	3.5 (1–4.5)	1
Revolutions per minutes	2,990 (2,500–3,450)	261.5
CRRT type and parameters		
CRRT (yes/no), n (%)	14/18 (43.7/56.3)	NA
CVVH, n (%)	11 (78.6)	NA
CVVHD, n (%)	1 (7.14)	NA
CVVHDF, n (%)	2 (14.3)	NA
Length of CRRT therapy (hours)	82 (8–627)	193
Blood flow rate (L/min)	150 (140–200)	10
Dialysate flow rate (mL/h)	2,000 (1,000–2,000)	500
Hemodilution parameters		
24 h input (mL)	2,620 (829–9,220.6)	1,520
24 h output (mL)	2,700 (201–10,841)	1,776
Clinical outcomes		
Length of ICU stay (days)	17.5 (4–97)	26
Length of hospital stay (days)	22 (4–99)	30
In-hospital mortality, n (%)	8 (25)	NA

Abbreviations: SD, standard deviation; IQR, interquartile range; APACHE, acute physiology and chronic health evaluation; COPD, chronic obstructive pulmonary disease; ECMO, extracorporeal membrane oxygenation; CRRT, continuous renal replacement therapy; CVVH, continuous veno-venous hemofiltration; CVVHD, continuous veno-venous hemodialysis; CVVHDF, continuous veno-venous haemodiafiltration; ICU, intensive care unit.

^a^ The data were presented as median (range) or frequency (percentage), unless indicated otherwise.

### 3.2 Population pharmacokinetic analysis

**FIGURE 3:**
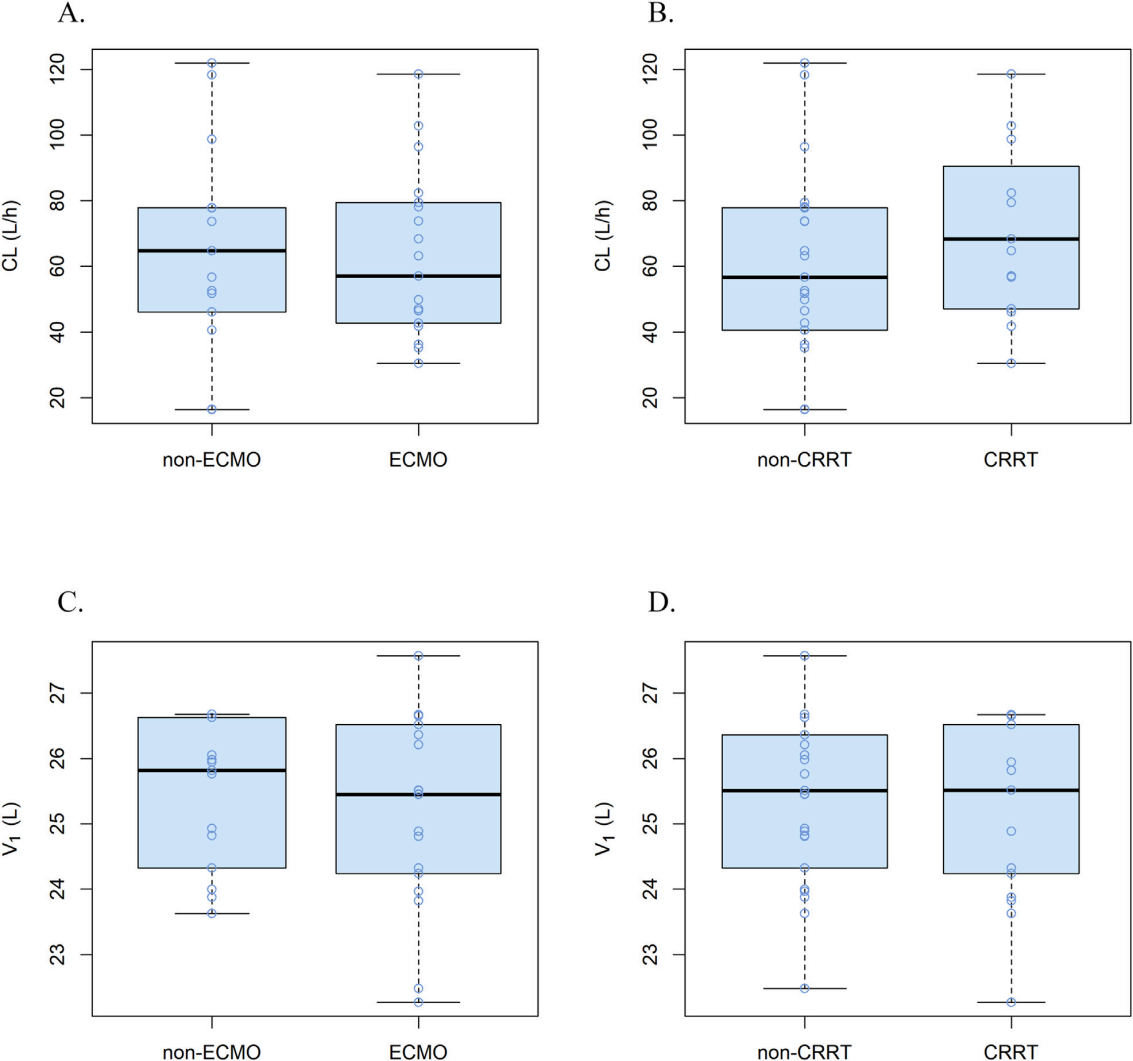
Correlations plots for covariates and pharmacokinetic parameters. **(A)** CL in ECMO vs non-ECMO patients, **(B)** CL in CRRT vs non-CRRT patients, **(C)** V1 in ECMO vs non-ECMO patients and **(D)** V1 in CRRT vs non-CRRT patients.

Parameter comparisons for the one-, two-, and three-compartment models were summarized in Supplementary Table S2. According to the goodness-of-fit plots and the precision of the parameter estimations, a two-compartment model appropriately described the concentration-time profile of remimazolam in critically ill patients, with a proportionality error of 25%. Table 2 shows the pharmacokinetic parameter estimates for the final model and 95% confidence intervals (CI) based on bootstraps. No covariates were found to influence significantly the pharmacokinetic parameters of remimazolam. Regardless of whether the patients were treated with ECMO or CRRT, there were no meaningful variations in the pharmacokinetic parameters (Figure 3). The procedure for screening of all covariates was shown in Supplementary Table S3. The elimination CL, volume of the central compartment (V_1_), volume of the peripheral compartment (V_2_), and peripheral compartmental clearance (Q) were estimated to be 58.2 L/h (95% CI, 47.8–72.3 L/h), 25.5 L (95% CI, 16.8–33.3 L), 34.5 L (95% CI, 26.0–58.8 L), and 21.9 L/h (95% CI, 12.2–34.6 L/h), respectively. Other model parameters, including rate constants and half-lives, were provided in Supplementary Table S4.

**TABLE 2:** Pharmacokinetic parameter estimates for the final model and bootstrap results.

Parameters	Base model		Median by 1,000 bootstraps	95% CI by 1,000 bootstraps
	Estimate (% RSE)	Shrinkage (%)		
Fixed effects				
CL (L/h)	58.2 (10.6)	NA	58.7	47.8–72.3
V_1_ (L)	25.5 (12.8)	NA	25.2	16.8–33.3
Q (L/h)	20.0 (21.8)	NA	21.9	12.2–34.55
V_2_ (L)	34.5 (17.0)	NA	37.5	26.0–58.8
Random effects				
IIV-CL (%)	50.3 (15.6)	1	24.7	11.4–41.4
IIV-V_1_ (%)	16.5 (68.7)	69	3	0.03–93.8
IIV-Q (%)	65.7 (50.0)	27	44.2	0.5–115.6
IIV-V_2_ (%)	61.2 (24.7)	15	29.9	10.6–60.2
Proportional error (%)	25.0 (13.0)	NA	23.5	18.1–29.6

Abbreviations: CL, clearance of the central compartment; V_1_, volume of the central compartment; Q, peripheral compartmental clearance; V_2_, volume of the peripheral compartment; IIV, the inter-individual variability.

**FIGURE 4:**
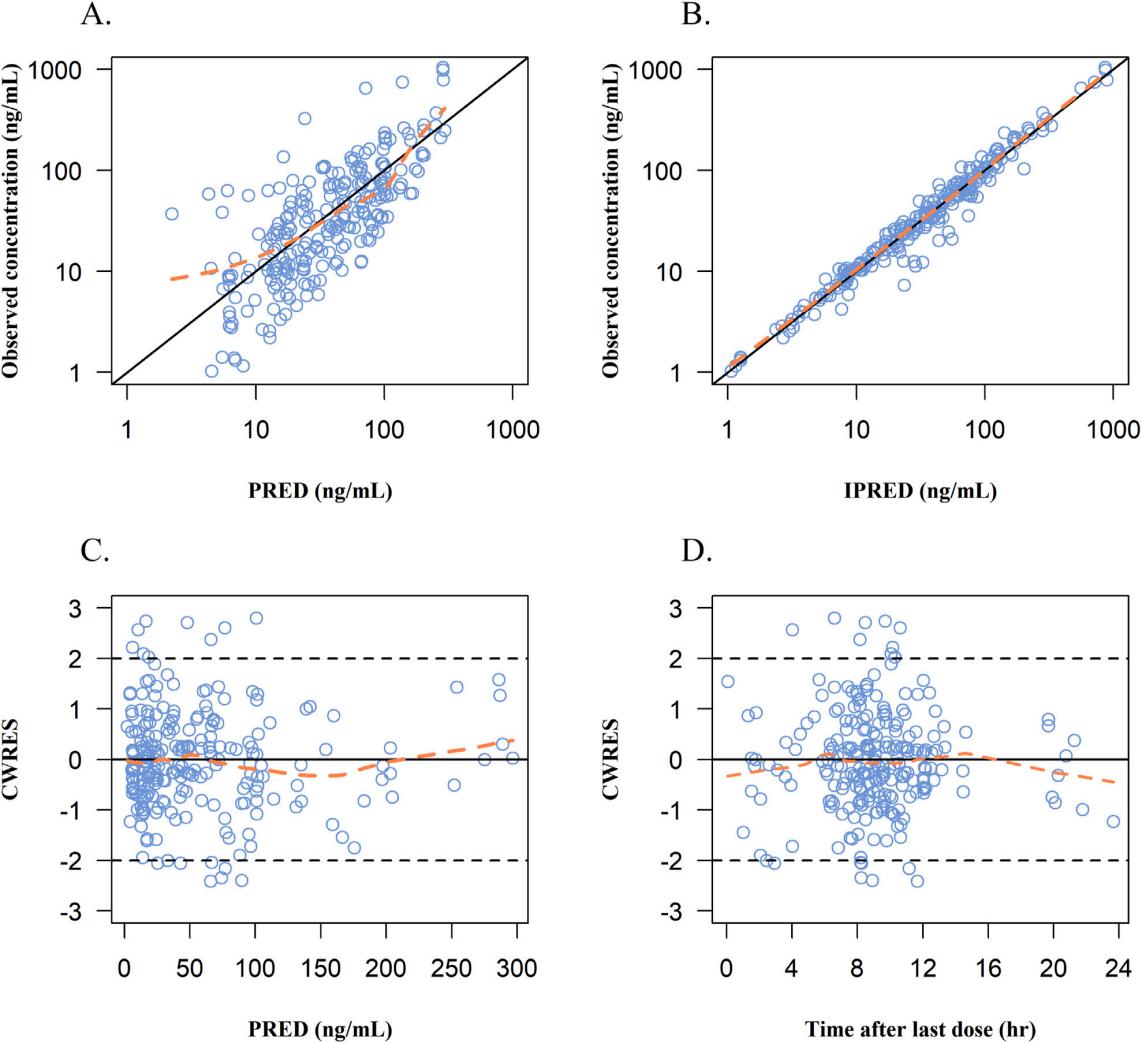
Goodness-of-fits plots for the final PopPK model. **(A)** Observed concentrations vs population prediction (PRED). **(B)** Observed concentrations vs individual predicted concentrations (IPRED). **(C)** Conditional weight residual error (CWRES) vs population predicted concentrations (PRED). **(D)** CWRES vs time after last dose. Blue dots represent remimazolam plasma concentrations; solid black line in **A and B**: line of identity; Orange line, data smoother; solid black line in **C and D**: CWRES are equal to 0.

**FIGURE 5:**
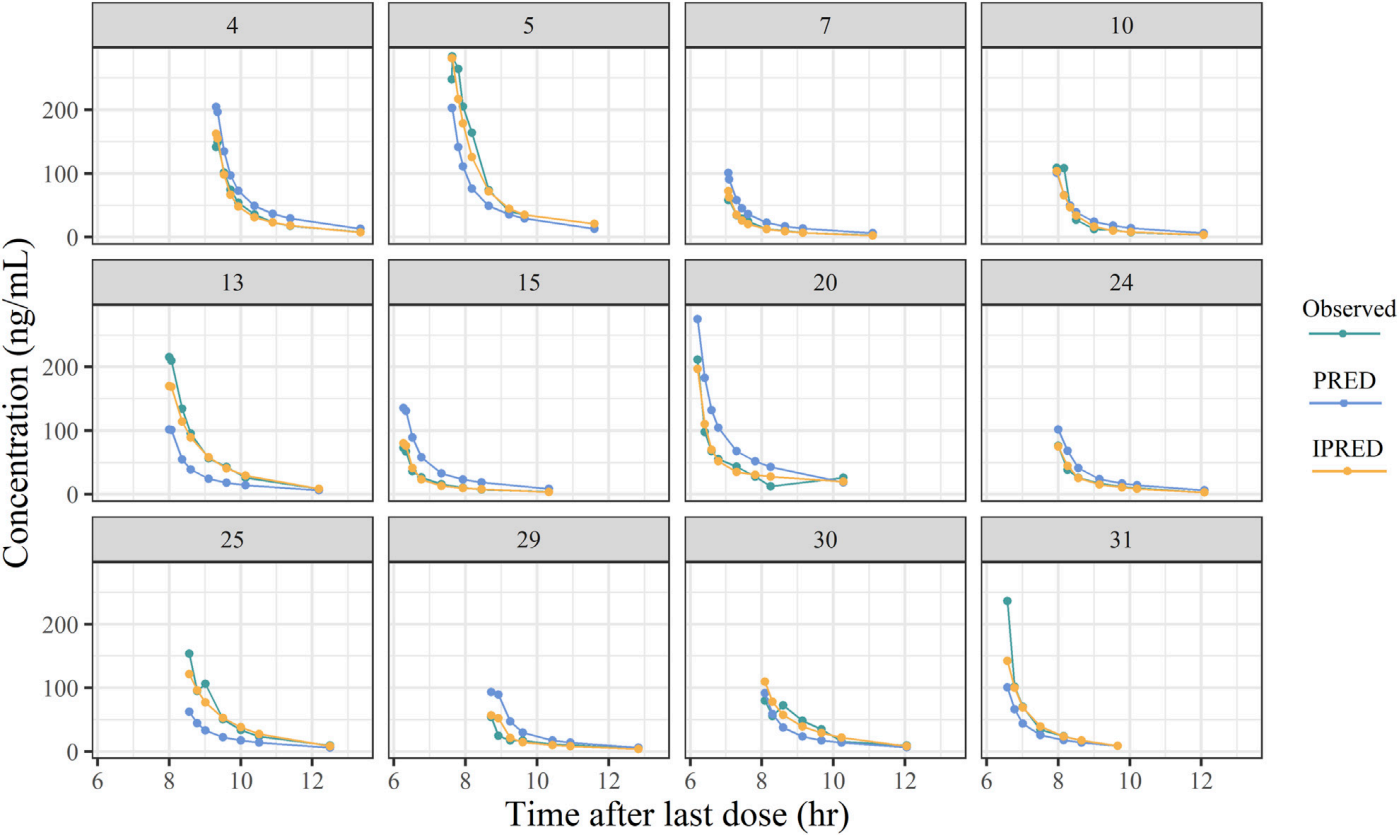
Plots of the typical individual plasma concentrations vs time after last dose.

The goodness-of-fit plots of the final model for remimazolam are shown in Figure 4. The observed concentrations (Figures 4A,B) were clustered in the proximity of the reference line (*y* = *x*), suggesting a well-fitted model. In the graphs of CWRED vs PRED and time after the last dose, most of the data were uniformly distributed on both sides of the reference line (*y* = 0) and within ±2, which indicated that the model had a good prediction performance. The individual concentration-time profiles (Figure 5) provided a visual assessment of the predictions of the model for each patient.

**FIGURE 6:**
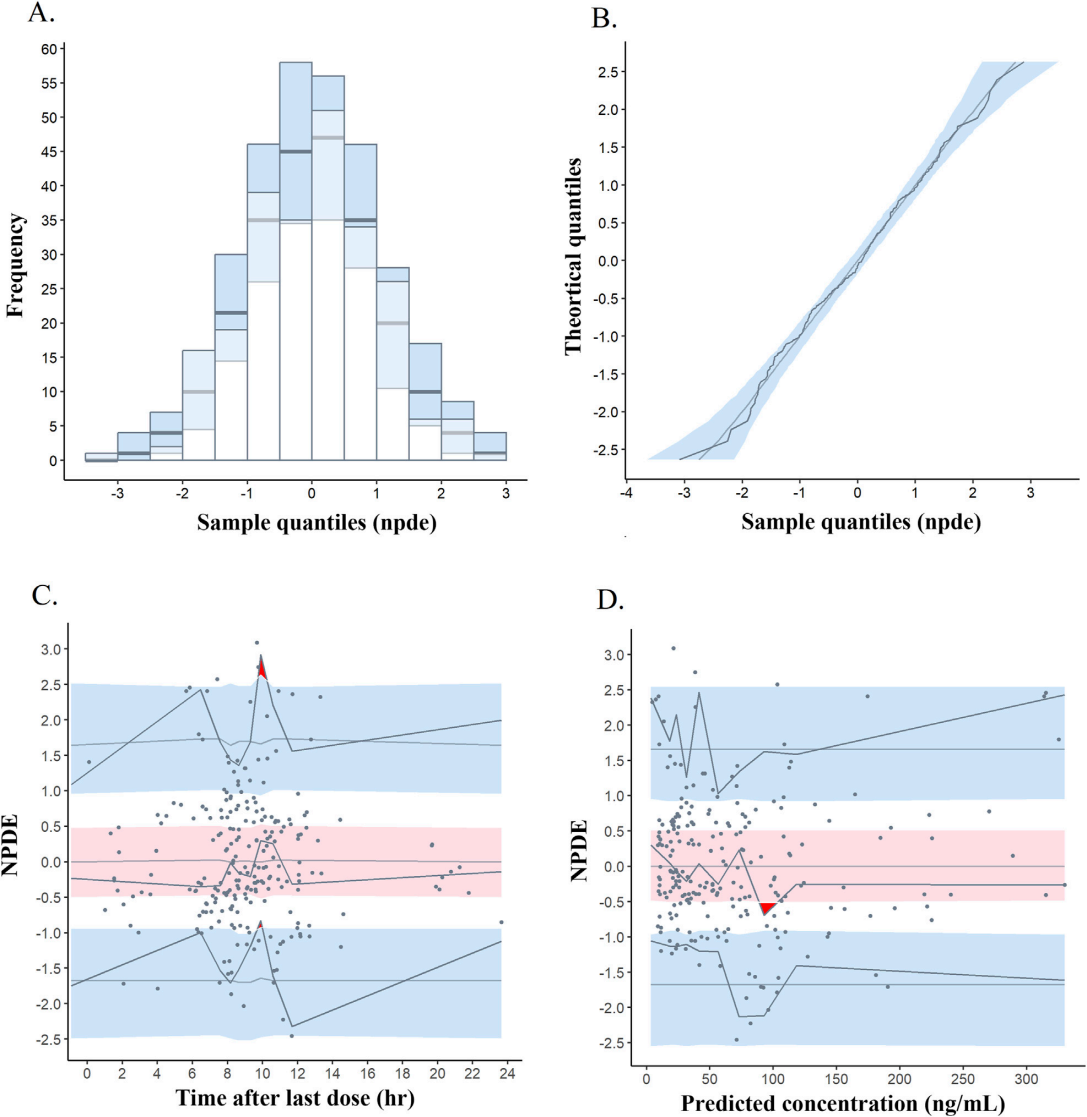
Normalized prediction distribution error (NPDE) metrics for the final PopPK model. Notes: **(A)** Histogram of the distribution of NPDE. **(B)** Quantile-quantile plot of NPDE. **(C)** NPDE vs time after last dose. **(D)** NPDE vs predicted concentrations. Solid points represent observed concentrations, and solid lines indicate the 5th, 50th, and 95th percentiles of observed data. Red or blue shaded areas represent 95% prediction intervals.

In pcVPC, most of the observations were within 90% of the prediction interval of the analogue data, which demonstrated that the model possessed an accurate prediction profile (Supplementary Figure S2). Through 1,000 simulations, the distribution of NDPE with a mean of −0.009 and a variance of 1.026, and results of the statistical tests revealed a normal distribution (Figures 6A,B). In the plots of NDPE vs time and population concentrations (Figures 6C,D), the values were found to be uniformly distributed between *y* = ± 2, indicating the validity of the final model.

### 3.3 Predicted concentration profiles

**FIGURE 7:**
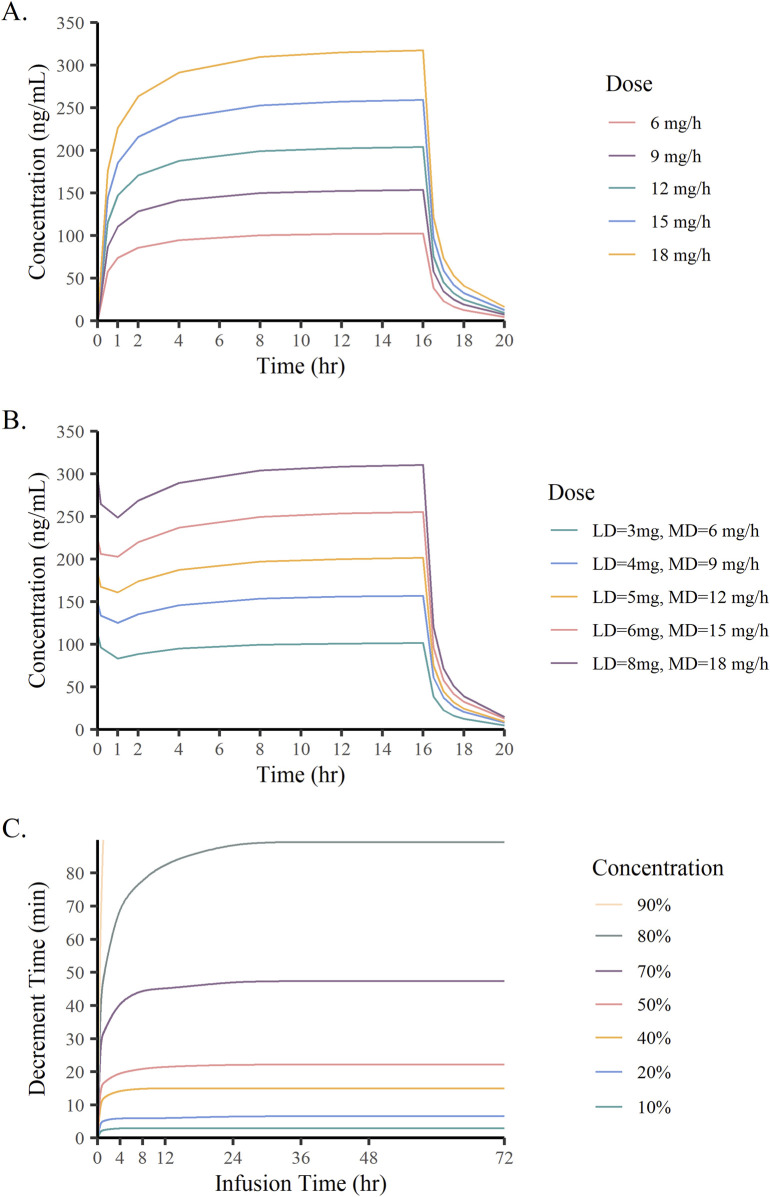
Monte Carlo simulation results. **(A)** Plasma concentrations of remimazolam administered at different constant rate; **(B)** Plasma concentrations of remimazolam at different loading and maintenance doses; **(C)** Time of remimazolam concentration decrement by 10%–90% vs infusion time.

Figures 7A,B show the concentration-time curves of remimazolam under different dose scenarios. When remimazolam was administered at a constant rate, it took about 4 h to achieve steady state blood concentrations. After an initial loading dose (3–8 mg) given within 1 min, the plasma concentration of remimazolam rapidly peaked and tended towards a steady-state at the maintenance dose (3–18 mg/h). Simulations revealed that different doses of remimazolam cleared rapidly after discontinuation of the infusion. The CSDTs for 10%–90% decrements in remimazolam concentrations are presented in Figure 7C. In curves with the concentrations decreasing from 10% to 50%, the CSDTs at 4 h and at later times trended to the same level. The CSHTs (the CSDTs for 50% decrement) was 15.6 min, 18 min and 21 min at 0.5 h, 2 h and 8 h, respectively, followed by little elevation. The results clearly demonstrated that CSHTs were independent of prolonged infusion.

## 4 Discussion

### 4.1 Findings and the impact of covariates

As far as we are aware, this is the first prospective study to use PopPK to analyze the pharmacokinetic properties of remimazolam in critically ill patients. The results showed that continuous infusion of remimazolam was not susceptible to accumulation in critically ill patients with complex pathophysiological states, such as abnormal hepatic or renal functions, or under ECMO and CRRT therapy. In the course of administration, the dosage was adjusted only according the sedative effect.

In previous PopPK studies (Schüttler et al., 2020; Zhou et al., 2020; Stöhr et al., 2021), three-compartments was primarily chosen as the base model. However, our study collected arterial blood samples by sparse blood sampling during the elimination phase after continuous micropump administration. And after comparing one-, two- and three-compartment models, it was found that the two-compartment model had a better fit and smaller precision for the pharmacokinetic parameters of critically ill patients. The decision to choose a different model can be influenced by variations in dosage schedules, sampling processes, research populations and analytical methods (Yang et al., 2017). The pharmacokinetic characteristics of healthy volunteers were investigated in the studies by Sheng (Sheng et al., 2020), Wiltshire (Wiltshire et al., 2012) and Schüttler (Schüttler et al., 2020) et al., whereas in the study by Zhou et al. (2020) in surgical subjects, the CL total ranged from 56.4 to 72 L/h. In our study, the CL was 58.2 L/h, which was similar to those published in previous studies, suggesting no significant effect of different populations on CL.

Weight seemed to be a key covariate in the exploration of the anesthesia agent, typically in relation to the dose administered. In a phase 1 study on healthy subjects (Antonik et al., 2012), it was concluded that weight (60–100 kg) had little effect on the systemic CL of remimazolam. Another study (Masui et al., 2022) that enrolled surgical patients found higher blood levels in heavier patients than in lighter ones when weight-dependent doses were administered. They suggested that weight-unadjusted dosage regimens may also be an option. Consistent with the above reports, the present study considered that weight ranging from 47 to 98 kg was not a significant covariate for the pharmacokinetic parameters of remimazolam. Practical dosing regimens based on clinical effect instead of body weight should be a more appropriate choice (Erstad and Barletta, 2020). The distribution of sample characteristics may also influence the results of the study. In this study, the weight range of the sample was predominantly in the 47–75 kg range, with only three patients exceeding this range, thus it may be difficult to extrapolate to the overweight population.

In a previous study (Masui et al., 2022) it was reported that a higher infusion rate of approximately 10% was necessary to maintain remimazolam concentrations in female patients. In performing the forward stepwise inclusion of covariates, gender had an effect on CL [a difference of OFV (dOFV) of −6.244, α < 0.05], and in a subsequent backward elimination the increase in dOFV was found to be <10.82 (α > 0.001), which failed to meet the final criteria for screening. In the light of previous studies, we simulated the effect of gender on concentration (Supplementary Figure S3) and found that the CL was 1.3-fold higher in females than in males. However, in the present study, the number of male patients was three folds that of female, which would have impacted on the reliability of the results and ultimately the covariate was discarded.

It is commonly reported (Freyer et al., 2019; Sneyd and Rigby-Jones, 2020) that the metabolism of remimazolam is primarily hydrolysis by CES-1, which is highly expressed in the liver (Carithers et al., 2015), and its hydrolysis products are excreted *via* the kidneys. Considering that there are considerable variations in hepatic and renal functions for critically ill patients, it was vital to assess biochemical indicators of hepatic and renal function. Thomas et al. studied patients with hepatic (n = 11) and renal (n = 11) impairment and found that exposure in patients with severe hepatic insufficiency (n = 3) was 38.1% higher than in healthy volunteers. In this study, covariates related to hepatic function including ALT, TP, ALB, TBIL and DBIL were investigated (Bernardi, n.d.). The results revealed an increase in V_1_ with elevated ALT, yet this finding was not incorporated into the final model due to the insufficient significance of covariate inclusion (α < 0.001). In Supplementary Figure S3, we have illustrated the influence of ALT on the concentration-time, confirming that the time for remimazolam CL was not significantly extended when ALT was higher than normal (0–40 U/L). The potential mechanism could be that CES-1 was highly expressed not only in the liver, but also in the gallbladder and lungs (Uhlén et al., 2015; Tissue expression of CES1 - Summary - The Human Protein Atlas, n. d.), so that a pathway for extra-hepatic esterase metabolism may exist. In addition, there appear to be no published data to support the view that the primary metabolic enzyme of remimazolam is CES-1 (Kilpatrick, 2021). As for renal function, the SCR, calculated CrCL and eGFR were analyzed, and consistent with the conclusions of Stöhr (Stöhr et al., 2021), no correlation was found with the pharmacokinetic parameters of remimazolam. Since the metabolite CNS-7054 exhibited no sedative activity (Kilpatrick et al., 2007), dosage adjustments were not required in patients with renal impairment.

ECMO is a life-support modality for patients with refractory cardiac and/or respiratory failure with complex pharmacokinetic profiles of drugs used during treatment (Ha and Sieg, 2017; Song et al., 2022). *In vitro* ECMO modelling revealed that drugs with lipophilic or highly protein-binding properties may interact with the circuit material and exhibit an increase in Vd or a change in CL (Dagan et al., 1993; Shekar et al., 2015). Given *in vitro* studies without blood, these may overestimate the adsorption effect in the ECMO circuit (Cheng et al., 2019), which requires more clinical trials to assess the impact of ECMO. Remimazolam with its lipophilic structure may be adsorbed by the ECMO circuit, therefore the present study analyzed the occurrence of ECMO treatment and the parameters of ECMO, including the duration of ECMO treatment, blood and gas flow and the pump speed. We enrolled 18 patients who received ECMO, but no significant influences on remimazolam CL or V_d_ were discovered. For patients requiring sedation with remimazolam during ECMO therapy, we concluded that a dose intervention was not required. In critically ill patients requiring CRRT, the physicochemical properties of the drug, acute pathophysiological variability, and CRRT settings may affect the achievement of the optimal pharmacokinetic/pharmacodynamic goals (Pistolesi et al., 2019; Hoff et al., 2020; Li et al., 2020). Our research indicated that the pharmacokinetic properties of remimazolam were unaffected by the presence of CRRT, the treatment duration, blood flow rate and dialysate flow rate. Remarkably, there was a great variation in CRRT modalities and settings (Gatti and Pea, 2021), the reasons for which need to be further explored.

### 4.2 Simulation

The rate of drug elimination is usually affected by the duration of anesthetic administration which influences the duration of the anesthetic effect (Schraag et al., 1998; Eger and Shafer, 2005). For intravenous anesthetics, computer simulations are commonly used to estimate the CSDTs, which is a valid measure to quantify the pharmacokinetics of drug concentration after continuous intravenous infusion (Bailey, 1995). Multiple decrement times (10%–90%) and longer infusion times (0.5–72 h) were simulated in the present study, and all curves plateaued after 24 h, with the CSHTs ranging from 15.6 to 21 min. In comparison with our results, a shorter of arterial plasma CSHT of 7–8 min was found in a study conducted by Wiltshire et al. (2012) of a remimazolam (50 mg/h) infusion for 1 min to 8 h. While Masui and colleagues study results (Masui, 2020) and Schüttler et al. (Schüttler et al., 2020) observations of CSDTs of 15.9–19.1 min and 12 ± 2 min for effect site concentrations after 4-h infusion were more comparable to our findings. We suspect that the two-compartment model used in this investigation made it difficult to distinguish between plasma and effect site (Hughes et al., 1992; Eger and Shafer, 2005). The results can be influenced by combination medication, patient characteristics and the remimazolam dose regimens (Masui, 2020). Of significance, the CSDTs of remimazolam did not correlate with the duration of the infusion, implying that remimazolam is appropriate for long-term administration to sedate patients in ICUs.

### 4.3 Pharmaceutical synergies

In the simulation of the dosing regimen, we noted that the routine dosage for ICU sedation produced blood concentrations that did not reach the sedation concentrations (400–1,200 ng/mL) suggested by Sheng et al. (2020) which may be related to the co-administration of remifentanil. It has been reported (Zhou et al., 2020) that combined remifentanil significantly reduced sedation scores, and almost all patients (96.3%) in our study were co-administered remifentanil. The study by Sheng et al. modeled blood concentrations of remimazolam based on BIS values, whereas Shirozu et al. (2022) found that BIS was relatively high during anesthesia with remimazolam. That explained the sufficient sedative effect of lower plasma concentration levels of remimazolam in the present study.

In summary, the pharmacokinetic profile exhibited by remimazolam was appropriate for ICU sedation. However, in a trial (Oh et al., 2023) of remimazolam vs propofol for general anesthesia, re-sedation within 30 min postoperatively was observed only in the remimazolam group [11 (22%) patients)] Of these, 1 patient was fully awake within a few min without the need for additional flumazenil, whereas the remaining 10 patients were treated with an additional dose of flumazenil (0.2 mg) and were fully awake within the next 15 min. Moreover, a case (Takemori et al., 2022) of long-term delayed emergence with re-sedation was reported and the patient stayed drowsy after three flumazenil administrations. A recent review that (Kempenaers et al., 2023) summarized the adverse events of remimazolam reported that the incidence of re-sedation may be directly related to flumazenil administration. It should be noted that the monitoring of clinical symptoms and blood concentrations after termination of a remimazolam infusion may be helpful in searching for the cause of re-sedation.

### 4.4 Limitations

Some limitations of the present study should be discussed. First, the fact that this was a single-center study with a small sample size may restrict the generalizability of the results and the capacity to evaluate the influence of variables on PopPK parameters. However, a total of 243 plasma samples were collected and were sufficient for establishing a PopPK model. Second, for critically ill patients, sedative and analgesic agents are generally required to be simultaneously administered (Vincent et al., 2016), making it problematic to implement the collection of pharmacodynamic data for remimazolam alone. As well, due to the restricted availability of metabolites of remimazolam, a controlled drug, it was not possible to establish a combined PopPK model for both compounds. Finally, there is controversy concerning liver functions (Stöhr et al., 2021). In our study, patients were not scored independently for liver functions, but rather they were assessed using biochemical indicators, which might not accurately reflect the connection between remimazolam metabolism and liver function. It is considered that future studies should focus on these aspects to evaluate their efficacy and safety for long-term sedation of ICU patients.

## 5 Conclusion

In conclusion, our research has established the first PopPK model for continuous intravenous infusion of remimazolam in critically ill patients. No covariates were observed that significantly influenced the pharmacokinetic parameters of remimazolam. Dose adjustments of remazolam are not necessary for patients undergoing ECMO or CRRT in the ICU, nor for those with impaired hepatic or renal function. Remimazolam with its predictable pharmacokinetic profile in this population is suitable for long-term sedation demands in the ICU and can be administered in accordance with the sedative effect without the necessity for significant dose adjustment.

## Data Availability

The original contributions presented in the study are included in the article/Supplementary Material, further inquiries can be directed to the corresponding author.
