# Supplementary material for: Population pharmacokinetic analysis of remimazolam after continuous infusion for sedation in critically ill patients
Source: Front Pharmacol. 2025 Jul 9;16:1526266. doi: 10.3389/fphar.2025.1526266 (PMC12283299; doi:10.3389/fphar.2025.1526266)
Supplement: Supplementary file 1 [file Supplementaryfile1.docx]

Supplementary Material

**Supplementary Table 1-** Physico-chemical properties of remimazolam

| **Parameter** | **​Value** |
| --- | --- |
| ​Chemical Formula | C_21_H_19_BrN₄O_2_ |
| ​Molecular Weight | 439.2 |
| ​Solubility | Benzenesulfonate remimazolam is soluble in water (0.1 g/mL) and organic solvents (e.g., methanol, acetone) |
| ​Stability | Stable at 5°C |
| ​Partition Coefficient | LogP = 2.1 (octanol/water) |

**Supplementary Table 2-** Result of PK parameter for one-, two- and three-compartment models

| **Parameters** | **One-compartment Model** | | **Two-compartment Model** | | **Three-compartment Model** | |
| --- | --- | --- | --- | --- | --- | --- |
|  | **Estimate** | **RSE (%)/**  **Shrinkage (%)** | **Estimate** | **RSE (%)/**  **Shrinkage (%)** | **Estimate** | **RSE (%)/**  **Shrinkage (%)** |
| **Fixed effects** |  |  |  |  |  |  |
| CL (L/h) | 68.6 | 10.5 | 58.2 | 10.6 | 53.3 | 10.9 |
| V1 (L) | 58.8 | 10.4 | 25.5 | 12.8 | 12 | 51.6 |
| Q (L/h) | - | - | 20 | 21.8 | 76.6 | 26.8 |
| V2 (L) | - | - | 34.5 | 17 | 26 | 21.7 |
| Q2 (L/h) | - | - | - | - | 7.54 | 22.8 |
| V3 (L) | - | - | - | - | 169 | 56.9 |
| **Random effects** |  |  |  |  |  |  |
| IIV-CL (%) | 50.4 | 2 | 50.3 | 1 | 53.6 | 1 |
| IIV-V1 (%) | 40 | 25 | 16.5 | 59 | 3.2 FIX | 97 |
| IIV-Q (%) | - | - | 65.7 | 27 | 3.2 FIX | 97 |
| IIV-V2 (%) | - | - | 61.2 | 15 | 62.7 | 15 |
| IIV-Q2 (%) | - | - | - | - | 11.1 | 30 |
| IIV-V3 (%) | - | - | - | - | 3.2 FIX | 98 |
| **Proportional error (%)** | 16.1 | 11.9 | 25 | 13 | 33.3 | 12.6 |
| **OFV** | 1613.15 | | 1462.256 | | 1429.032 | |

**Supplementary Table 3-** Forward stepwise univariate analysis process for screening covariates

| **Model** | **Covariates** | **OFV** | **ΔOFV** | **p value** |
| --- | --- | --- | --- | --- |
| Base | Two-compartment model | 1462.256 | - |  |
| 1 | CL-SEX | 1456.012 | -6.244 | <0.01 |
| 2 | V1-SEX | 1456.219 | -6.037 | <0.01 |
| 3 | CL-WEIGHT | 1457.646 | -4.61 |  |
| 4 | V1-WEIGHT | 1462.256 | 0 |  |
| 5 | CL-AGE | 1456.692 | -5.564 |  |
| 6 | V1-AGE | 1457.646 | -4.61 |  |
| 7 | CL-BMI | 1457.646 | -4.61 |  |
| 8 | V1-BMI | 1457.646 | -4.61 |  |
| 9 | CL-CRRT | 1462.03 | -0.226 |  |
| 10 | V1-CRRT | 1462.136 | -0.12 |  |
| 11 | CL-ECMO | 1462.152 | -0.104 |  |
| 12 | V1-ECMO | 1462.033 | -0.223 |  |
| 13 | CL-SCR | 1460.623 | -1.633 |  |
| 14 | V1-SCR | 1456.145 | -6.111 | <0.01 |
| 15 | CL-ALT | 1457.646 | -4.61 |  |
| 16 | V1-ALT | 1450.889 | -11.367 | <0.001 |
| 17 | CL-TP | 1457.646 | -6.441 | <0.01 |
| 18 | V1-TP | 1462.256 | 0 |  |
| 19 | CL-ALB | 1457.646 | -4.61 |  |
| 20 | V1-ALB | 1457.146 | -5.11 |  |
| 21 | CL-TBIL | 1462.196 | -0.06 |  |
| 22 | V1-TBIL | 1462.256 | 0 |  |
| 23 | CL-DBIL | 1457.649 | -4.607 |  |
| 24 | V1-DBIL | 1462.256 | 0 |  |
| 25 | CL-URICACID | 1462.256 | 0 |  |
| 26 | V1-URICACID | 1452.405 | -9.851 | <0.01 |
| 27 | CL-CrCL | 1462.256 | 0 |  |
| 28 | V1-CrCL | 1462.256 | 0 |  |
| 29 | CL-CKDEPI | 1462.638 | 0.382 |  |
| 30 | V1-CKDEPI | 1462.256 | 0 |  |
| 31 | CL-WBC | 1457.646 | -4.61 |  |
| 32 | V1-WBC | 1457.646 | -4.61 |  |
| 33 | CL-PCT | 1461.36 | -0.896 |  |
| 34 | V1-PCT | 1462.638 | 0.382 |  |
| 35 | CL-LAC | 1457.646 | -4.61 |  |
| 36 | V1-LAC | 1454.585 | -7.671 | <0.01 |
| 37 | CL-NA | 1459.508 | -2.748 |  |
| 38 | V1-NA | 1457.646 | -4.61 |  |
| 39 | CL-K | 1457.646 | -4.61 |  |
| 40 | V1-K | 1456.942 | -5.314 |  |
| 41 | CL-PH | 1462.271 | 0.015 |  |
| 42 | V1-PH | 1457.646 | -4.61 |  |
| 43 | CL-PLT | 1462.265 | 0.009 |  |
| 44 | V1-PLT | 1456.831 | -5.425 |  |
| 45 | CL-HGB | 1457.646 | -4.61 |  |
| 46 | V1-HGB | 1457.646 | -4.61 |  |
| 47 | CL-HCT | 1457.646 | -4.61 |  |
| 48 | V1-HCT | 1457.646 | -4.61 |  |
| 49 | CL-CYCS | 1457.446 | -4.81 |  |
| 50 | V1-CYCS | 1457.646 | -4.61 |  |
| 51 | CL-CRP | 1455.504 | -6.752 | <0.01 |
| 52 | V1-CRP | 1462.256 | 0 |  |
| 53 | CL-BUN | 1458.578 | -3.678 |  |
| 54 | V1-BUN | 1457.646 | -4.61 |  |

**Supplementary Table 4-Parameters of the two-compartment model**

| **Parameter** | **Estimate** | **95%CI** |
| --- | --- | --- |
| **Rate Constants** | | |
| k10 (h⁻¹) | 2.28 | 1.44-4.3 |
| k12 (h⁻¹) | 0.78 | 0.37-2.06 |
| k21 (h⁻¹) | 0.58 | 0.21-1.33 |
| **Half-Lives** | | |
| t1/2a (h) | 0.21 | 0.1-0.38 |
| t1/2β (h) | 1.7 | 0.83-4 .3 |


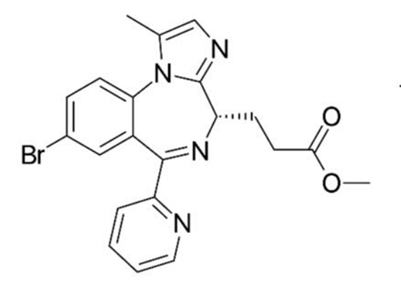


**Supplementary Figure 1.** The schematic diagram of the chemical structure of remimazolam.


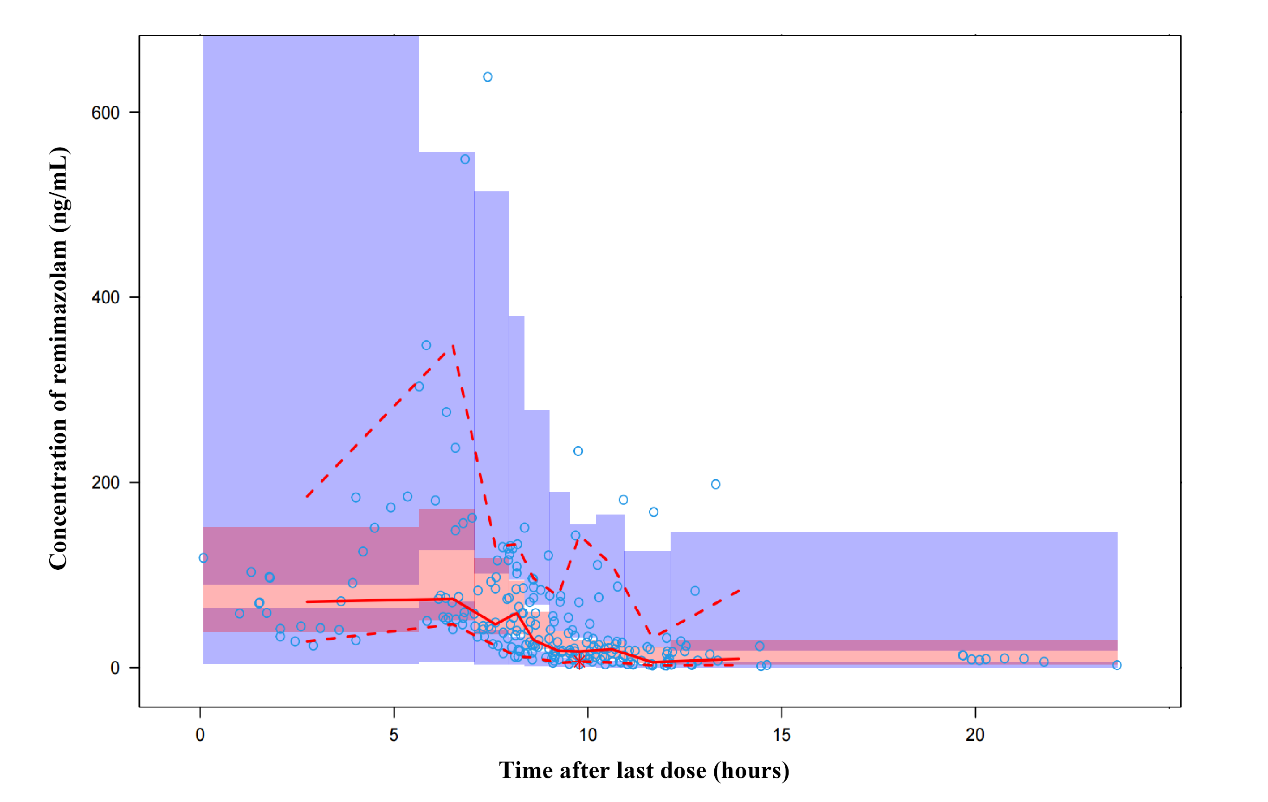


**Supplementary Figure 2.** Prediction-corrected visual predictive check for the final PopPK model of remimazolam. Solid red line lines represented the 50th percentile of observed data, dashed red lines represented the 5th and 95th percentiles of observed data. Red or blue shaded areas represent the 90% prediction interval.


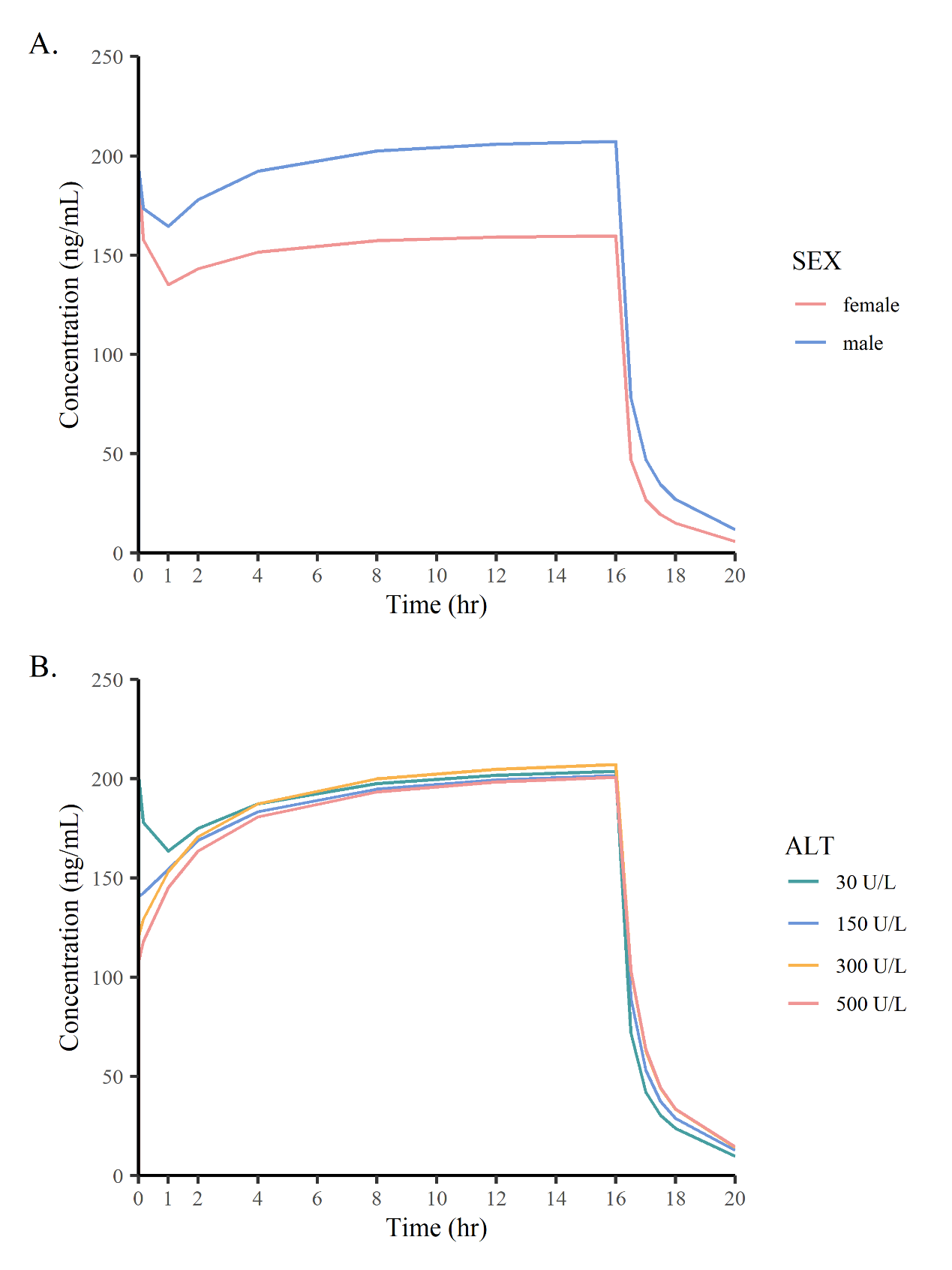


**Supplementary Figure 3.** Simulation results of model with sex and ALT included as covariates.
